# Collective Perception Using UAVs: Autonomous Aerial Reconnaissance in a Complex Urban Environment

**DOI:** 10.3390/s20102926

**Published:** 2020-05-21

**Authors:** Petr Stodola, Jan Drozd, Karel Šilinger, Jan Hodický, Dalibor Procházka

**Affiliations:** 1 Department of Intelligence Support, University of Defence, 66210 Brno, Czech Republic; 2 Department of Tactics, University of Defence, 66210 Brno, Czech Republic; jan.drozd@unob.cz; 3 Department of Fire Support, University of Defence, 66210 Brno, Czech Republic; karel.silinger@unob.cz; 4 NATO Headquarters Supreme Allied Commander Transformation, Norfolk, VA 23551, USA; jan.hodicky@act.nato.int; 5 Centre for Security and Military Strategic Studies, University of Defence, 66210 Brno, Czech Republic; dalibor.prochazka@unob.cz

**Keywords:** autonomous aerial reconnaissance, collective perception, metaheuristic algorithm, simulated annealing, experiments

## Abstract

This article examines autonomous reconnaissance in a complex urban environment using unmanned aerial vehicles (UAVs). Environments with many buildings and other types of obstacles and/or an uneven terrain are harder to be explored as occlusion of objects of interest may often occur. First, in this article, the problem of autonomous reconnaissance in a complex urban environment via a swarm of UAVs is formulated. Then, the algorithm based on the metaheuristic approach is proposed for a solution. This solution lies in deploying a number of waypoints in the area of interest to be explored, from which the monitoring is performed, and planning the routes for available UAVs among these waypoints so that the monitored area is as large as possible and the operation as short as possible. In the last part of this article, two types of main experiments based on computer simulations are designed to verify the proposed algorithms. The first type focuses on comparing the results achieved on the benchmark instances with the optimal solutions. The second one presents and discusses the results obtained from a number of scenarios, which are based on typical reconnaissance operations in real environments.

## 1. Introduction

Recently, swarm robotics has become a phenomenon in many real-world applications. It is about the coordination of multiple robots as a system to achieve a desired behavior. Collection of information about the environment is the most critical prerequisite for decision-making. For this, Unmanned Aerial Vehicles (UAVs) equipped with necessary sensors may be used in many situations and applications both in the civil and military domains.

In this article, the problem of using a swarm of UAVs in military reconnaissance operations is formulated and the solution to this problem is proposed. The principles of collective perception are used during the process in order to accelerate the operation and increase its effectiveness. The reconnaissance operations are assumed to be performed in complex urban environments and/or very uneven terrain where obstacles or terrain may cause occlusions.

The article is organized as follows: in this section, the authors’ motivation is discussed along with the implementation of the results. Contributions of the research are also highlighted. Section 2 reviews the literature related with these issues. Section 3 defines and formulates the problem. In Section 4, algorithms for a solution are proposed. Section 5 presents the results of experiments carried out for verification. Finally, Section 6 concludes the article and points out some possibilities of the future research in this area.

### 1.1. Motivation

The Military Decision-Making Process (MDMP) is one of the crucial processes in order to accomplish all the given tasks and missions. It is a very complex process composed of several steps. In the modern environment, any commander’s decision requires an analysis of countless information from different sources. Contemporary armed conflicts are characterized by a “big data” flow and its analysis, which significantly influences the MDMP. Moreover, the accuracy of any data used within the MDMP would tremendously influence the planning, development and subsequent evaluation of military missions. In order to support military commanders’ decision-making, the University of Defense developed the Tactical Decision Support System (TDSS) composed of several models of military tactics. It is designed as an open system with the possibilities of further development.

Reconnaissance itself is a very important step of the MDMP, especially on the tactical level. It provides military commanders with essential information concerning the terrain, weather, enemy situation, etc. It should be continuous and as much accurate as possible. Especially aerial reconnaissance conducted by UAVs is a very strong means of information gathering. Moreover, its autonomy did not require any additional demand on the military structure and organization.

The urban environment is currently one of the typical areas where contemporary armed conflicts take place. The armed conflicts in Syria, Ukraine and other places proved the necessity to deeply develop the information flow. The complexity of urban areas and different military tactics used require new approaches to support the MDMP of commanders. Subsequently, such new approaches would influence military tactics and diminish losses. There are several publications focused on this issue from different perspectives [1,2,3,4,5,6].

The model proposed in this article has been implemented as a part of the TDSS. The purpose of this system is to support commanders of the Czech Army on the tactical level. The implementation is still only in its trial version; this means it has been validated so far only by simulations. The real life experiments using drones in the real environment are still to be conducted. The idea of using this system for commander’s decision-making support is as follows. The commander, who needs to perform reconnaissance of some area of interest within his/her mission, inputs all necessary information and requirements into the system. Then, the system plans the routes of available UAVs at the disposal of the commander and presents the results for confirmation. Finally, the commander decides whether to launch the reconnaissance as he/she is responsible for the mission. After this decision, the reconnaissance operation can start immediately (or when required) by controlling the drones.

### 1.2. Contributions

The authors continue with their previous research in this area, see [7,8]. This article extends and completes their previous findings. In particular, the contributions are as follows:

- The problem of autonomous aerial reconnaissance in complex environments via a swarm of unmanned aerial vehicles is formulated. The monitoring is performed from a number of waypoints deployed in the area of operations; each waypoint is defined in the three-dimensional space by its coordinates and altitude.
- The approach to effectively evaluate the coverage (monitored area) from a number of deployed waypoints is proposed. Terrain and obstacles, which may occlude the objects of interest are taken into consideration as well as the parameters of the sensors used.
- The algorithm to estimate a minimum number of waypoints needed to explore a required portion of the area of interest is proposed.
- The metaheuristic algorithm based on the simulated annealing principles is proposed to deploy waypoints in the area of operations (in three dimensions).
- A set of experiments is designed to assess the proposed algorithms. The results are compared to the optimal solutions.
- A set of scenarios is designed based on the parameters and features of the real typical reconnaissance operations, and the behavior and results are discussed. The real geographic data is used in these experiments.

## 2. Literature Review

The topic concerning autonomous aerial reconnaissance using UAVs is currently discussed very frequently by the scientific as well as military personnel. From the scientific point of view, the reconnaissance problem, as is understood in this article, can be seen as the Art Gallery Problem, which is a well-studied NP-hard visibility problem in computational geometry [9]. This problem has been studied mostly in the two-dimensional space [10,11].

The three-dimensional case of the problem (referred to as 3D Art Gallery Problem) is studied less frequently. For example, Marzal [12] aimed at the determination of the number of guards required to cover the interior of a pseudo-polyhedron as well as the placement of these guards; this study models the art gallery by an orthogonal pseudo-polyhedron. Savkin and Huang [13] estimated the minimal number of drones necessary to monitor a given area of a very uneven terrain. The proposed problem may be viewed as a drone version of the 3D Art Gallery Problem. Thanou and Tzes [14] addressed the area coverage problem of a 3D-space by a group of UAVs, equipped with omnidirectional cameras; the terrain is assumed known to each UAV which has a maximum flight-altitude.

Some of the publications dealing with the problem of using UAVs for monitoring or surveillance take into consideration the fact that the area, where reconnaissance is carried out, is full of visual obstacles, particularly in an urban area. Saripalli et al. [15] presented the design and implementation of a real-time vision-based approach to detect and track features in a structured environment using an autonomous helicopter. Shaferman and Shima et al. [16] examined the problem of tracking the moving ground target in an urban environment via a set of cooperating UAVs and proposed a stochastic search method (based on a genetic algorithm) for finding in real time monotonically improving solutions. Semsch et al. [17,18] dealt with the problem of multi-UAV-based surveillance in complex urban environments with occlusions. The problem lies in controlling the flight of UAVs with on-board cameras so that the coverage and recency of the information about the designated area may be maximized. Swarming of multiple UAVs in order to increase the performance and effectiveness of the operation has been recently studied in many researches including applications such as reconnaissance or surveillance. Alfeo et al. [19] proposed a swarm coordination bio-inspired algorithm and applied it to search for a target object using a swarm of UAVs equipped with imperfect sensors. Similarly, Li et al. [20] introduced a distributed algorithm for searching moving targets via a fleet of cooperative UAVs. Silva et al. [21] examined the problem of real-time object identification and tracking through cooperative UAVs in a complex and adversarial environment involving motion, crowded scenes and varied camera angles and proposed a distributed deep learning algorithm for a solution.

Effective route planning and trajectory optimization of UAVs are critical in applications such as monitoring, reconnaissance or surveillance. Extensive surveys of planning methods are provided, for example, by Cabreira et al. [22] (methods based on coverage path planning), Zhao et al. [23] (computational-intelligence-based methods), Coutinho et al. [24] (identification of common attributes in aerial planning problems), or Geiger [25] (methods such as linear programming, dynamic programming, genetic algorithms and neural networks). In their research, Reardon and Fink [26] connected the issues of aerial reconnaissance (3D Art Gallery Problem) and path planning (Traveling Salesman Problem) and formulated the problem of searching and identification of objects using both ground and aerial autonomous robotic systems.

For the solution of the problem formulated in this article, two metaheuristic approaches were adapted: the simulated annealing for the waypoints deployment and the ant colony optimization for the path planning of UAVs. The choice of these methods has been supported by the experience of authors obtained in their previous research. Metaheuristic methods are, in general, problem dependent. Further on in this paragraph, several other methods, which could possibly be used to this problem, are mentioned on examples of their recent applications which are related to some extent to the problem examined in this article. A very popular method called Particle Swarm Optimization (PSO) is often used for various types of problems. It is a population based stochastic optimization technique developed in 1995 by Eberhart and Kennedy [27]. Since that time, a thousand of application have emerged. Shao et al. [28] use this method for the problem of generating cooperative feasible paths for formation rendezvous of UAVs which was formulated as a multi-objective optimization problem with many coupled constraints; the proposed algorithm can meet the kinematic constraints of UAVs and the cooperation requirements. Another very popular search heuristic approach belongs to the family of Genetic Algorithms (GA). It is inspired by Darwin’s theory of natural evolution; it reflects the process of natural selection. Cao et al. [29] adopted this algorithm for the problem of multi-base multi-UAV cooperative reconnaissance path planning; the problem is transformed into the shortest path combinatorial optimization using graph theory. However, the authors do not assume the occlusion caused by the terrain or obstacles. Tabu search formulated in 1986 by Glover [30] is another well-known local search method. Lee, Chen and Lai [31] hybridized this method with the 2-opt algorithm in the problem of the mission route planning of multiple unmanned robots in order to distribute tasks and coordinate the operation. A large family of bio-inspired algorithms has become very popular in recent years. To name a few: Artificial Bee Colony [32] (problem of UAVs used for wireless communication networks); Bat Algorithm [33] (problem of tracking a dynamic invading target by an UAV); or Grey Wolf Optimization [34] (multi-UAV multi-target urban tracking problem).

## 3. Problem Definition

In this section, the problem examined in this article is formulated. The problem is about planning routes of available UAVs for the reconnaissance mission performed in a complex urban environment. The goal is to plan the route so that the mission is carried out as fast as possible while the exploration of the area of interest is as complete as possible. The terrain and obstacles which may cause occlusions are taken into consideration. The list of all symbols and variables used in this and the following sections is included in the table at the end of the article.

Let $U=\left\{U_{1},U_{2},\ldots ,U_{M}\right\}$ be a finite set of UAVs where M≥1 is their number available to participate in a reconnaissance operation. At the beginning of the operation, each UAV is deployed in its initial position (base) in the area of operations. The UAVs launch from their bases, and, when the operation is over, they return back. Each UAV is equipped with a sensor system capable to monitor some portion of the area directly below it. These sensors are characterized by two parameters as follows:

- Angular field of view ($\alpha_{fov}$).
- Maximum distance from monitored objects ($d_{max}$).

**Figure 1:**
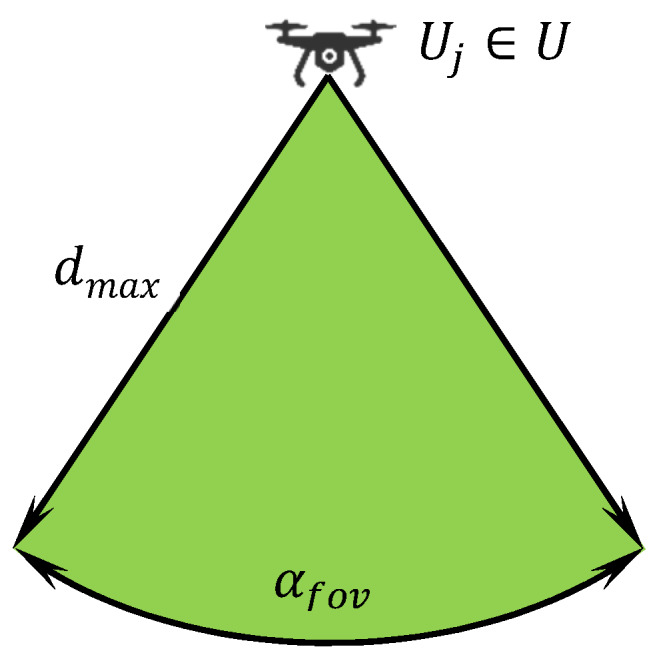
Object detection range of a sensor of an Unmanned Aerial Vehicles (UAV).

Both parameters are given by the technical construction and principles of the sensors. The sensors are homogeneous, i.e., all UAVs are equipped with the same type of sensors. Each UAV is able to monitor objects of interest located within the field of view of its sensor and not farther from the UAV than allowed by the maximum distance constraint. This principle in a plane is shown in Figure 1 where the green area (called object detection range) represents the space, in which the object, if located in this range, is detected by an UAV.

The aim of the reconnaissance operation is to monitor as much portion of the area of interest AoI as possible using a fleet of UAVs U. The area of interest is defined by a polygon with or without holes lying in the area of operations AoO (AoI⊆AoO). During the operation, the height of flight of the UAVs is restricted. The limiting parameters are as follows:

- Minimum height of flight above the ground level ($h_{min}$).
- Maximum height of flight above the ground level ($h_{max}$).

This means that the height of flight above the ground level h of all UAVs at any moment of the operation must be within the allowed limits ($h_{min}\leq h\leq h_{max}$). Both limits are set by the commander based on the tactical requirements for the operation and/or technical parameters of sensors.

Let $W=\left\{W_{1},W_{2},\ldots ,W_{N}\right\}$ be a finite set of waypoints deployed in the area of operations where N≥1 is their number. The monitoring of the area of interest is performed from these waypoints only. When any UAV is located at waypoint $W_{i}\in W$ at any time of the operation, some portion of the area of interest is observed (i.e., objects of interest are detected if located in the observed area).

Let $P=\left\{P_{1},P_{2},\ldots \right\}$ be an infinite set of points lying on the ground in the area of operations. Each point $P_{i}\in P$ is characterized by its elevation $E\left(P_{i}\right)$ (height above the mean sea level) determined by the terrain. An UAV monitoring from waypoint $W_{i}\in W$ is located at some altitude $h_{alt}^{i}$; see Equation (1) where $h_{i}$ is the height above the ground level of the UAV at waypoint $W_{i}$:

(1) $$h_{alt}^{i}=E\left(W_{i}\right)+h_{i}\;\;\;\mathrm{for}\;\mathrm{all}\;i=1,2,\ldots ,N$$

**Figure 2:**
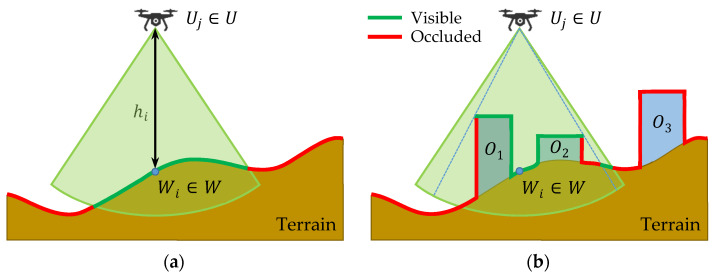
Determination of points monitored by an UAV: (**a**) without obstacles; (**b**) with obstacles.

Let $V_{i}\subseteq P$ be a set of points lying in the area of interest, which is monitored from waypoint $W_{i}$; this means that an object of interest is detected by an UAV positioned at waypoint $W_{i}$ and altitude $h_{alt}^{i}$ if this object is located at any point from set $V_{i}$. All points in $V_{i}$ must satisfy the conditions as follows:

1. Points in $V_{i}$ must lie in the area of interest ($V_{i}\subseteq AoI$).
2. Points in $V_{i}$ must be within the object detection range of the sensor (see Figure 1).
3. There must be a visual line of sight (VLOS) between the sensor and all points in $V_{i}$ (see Figure 2).

Let $O=\left\{O_{1},O_{2},\ldots ,O_{L}\right\}$ be a finite set of not-transparent obstacles lying in the area of interest where L≥0 is their number. An obstacle (e.g., a building) or an uneven terrain may occlude some portion of the area in the object detection range; this happens in case that the VLOS condition in point 3 above is violated. The principle in a plane is shown in Figure 2: Figure 2a shows the situation without obstacles, whereas Figure 2b with obstacles. The green line represents points in set $V_{i}\subseteq P$ (for further reference called as visible), red line remaining points in P but not in $V_{i}$ (for further reference called as occluded).

**Figure 3:**
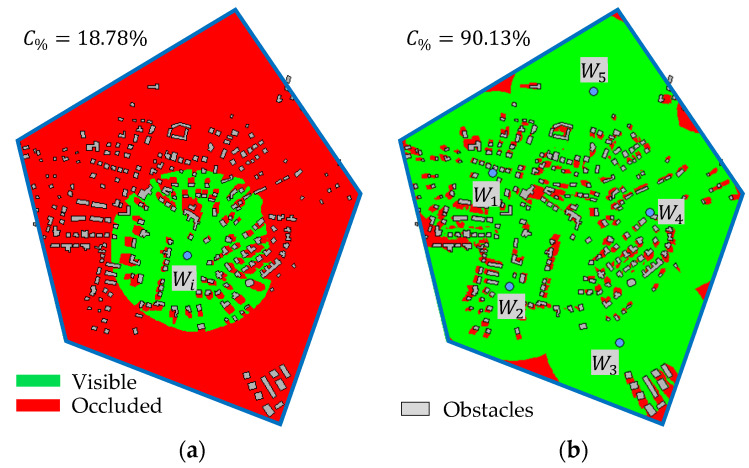
Visible points in the area of interest: (**a**) single waypoint; (**b**) multiple waypoints.

Figure 3a shows an example of the real situation from the top view. The polygon (blue line) encloses the area of interest; grey objects are obstacles (buildings in this case). Green area represents visible points in set $V_{i}$. Figure 3b shows the same situation, but this time, five waypoints are deployed. The total visible area V is given by the unification of sets $V_{i}$ for individual waypoints according to Equation (2). The total coverage of the area of interest through deployed waypoints is calculated according to Equation (3) as a ratio between the number of visible points (denoted as |V|) and the number of all points in the area of interest (denoted as |AoI|):

(2) $$V=\overset{N}{\underset{i=1}{\cup }}V_{i}$$

(3) $$C=\frac{\left|V\right|}{\left|AoI\right|}$$

During the reconnaissance operation, UAVs in the fleet visit individual waypoints in the specified order. As all sensors of the UAVs are homogeneous, it is irrelevant, which UAV will visit which waypoint. Let $R=\left\{R_{1},R_{2},\ldots ,R_{M}\right\}$ be a set of routes of individual UAVs. Route $R_{j}\in R$ defines a trajectory of UAV $U_{j}\in U$ as an order of waypoints to be visited: $R_{j}=\left\{R_{j}^{0},R_{j}^{1},R_{j}^{2},\ldots ,R_{j}^{K_{j}},R_{j}^{K_{j}+1}\right\}$ where $K_{j}\geq 0$ is the number of waypoints to be visited by UAV $U_{j}$. Constraints in Equations (4)–(7) need to be satisfied: constraint in Equation (4) ensures that each UAV launches from its base and returns back at the end of the operation; and constraints in Equations (5)–(7) ensure that all the waypoints are visited just once:

(4) $$R_{j}^{0}=R_{j}^{K_{j}+1}=U_{j}\;\;\;\mathrm{for}\;\mathrm{all}\;j=1,2,\ldots ,M$$

(5) $$R_{j}^{k}\in W\;\;\;\mathrm{for}\;\mathrm{all}\;j=1,2,\ldots ,M\;\mathrm{and}\;k=1,2,\ldots ,K_{j}$$

(6) $$\overset{M}{\underset{j=1}{\sum }}K_{j}=N$$

(7) $$R_{j}^{k}\neq R_{p}^{q}\;\;\;\mathrm{for}\;\mathrm{all}\;j,p=1,2,\ldots ,M\;(j\neq p),\;k=1,\;2,\ldots ,K_{j}\;\mathrm{and}\;q=1,\;2,\ldots ,K_{p}$$

Equation (8) defines the time, in which UAV $U_{j}$ performs its route $R_{j}$. The term $\left|R_{j}^{k}-R_{j}^{k+1}\right|$ expresses the time needed to fly from waypoint/base $R_{j}^{k}$ to the next waypoint/base $R_{j}^{k+1}$ on its route; it depends on the distance between both points and the flight parameters of UAV $U_{j}$. The duration of the reconnaissance operation is defined in Equation (9); all UAVs start at the same time at the beginning of the operation, and the operation ends when the last UAV returns back to its base:

(8) $$T_{j}=\overset{K_{j}}{\underset{k=0}{\sum }}\left|R_{j}^{k}-R_{j}^{k+1}\right|$$

(9) $$T=\max\left(T_{1},T_{2},\ldots ,T_{M}\right)$$

**Figure 4:**
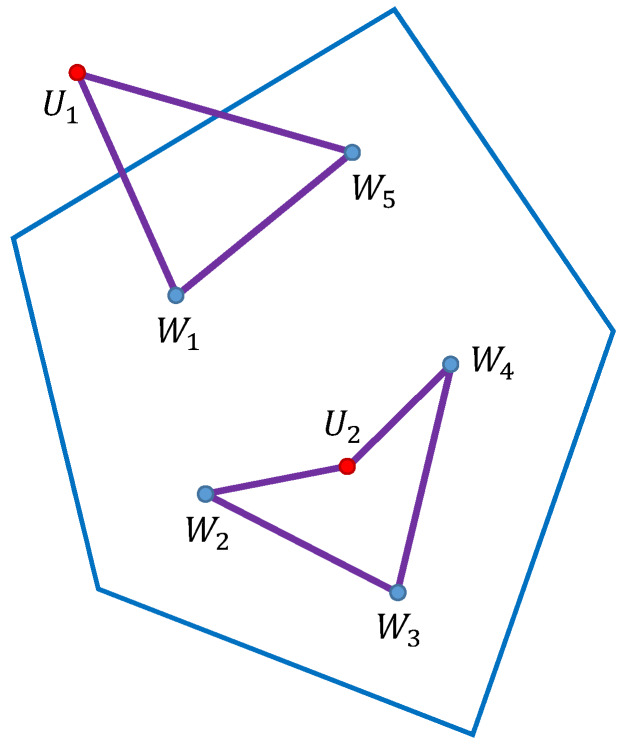
Routes of UAVs in the operation.

A simple example with two UAVs (red dots) and five waypoints (blue dots) is shown in Figure 4. The routes (violet color) are $R_{1}=\left\{U_{1},W_{1},W_{5},U_{1}\right\}$ and $R_{2}=\left\{U_{2},W_{2},W_{3},W_{4},U_{2}\right\}$.

It is desired to plan the reconnaissance operation optimally. The optimization criteria are defined in Equations (10)–(12). The first optimization criterion in Equation (10) is connected with the second optimization criterion in Equation (11). The former minimizes the number of waypoints N (the goal is to find such a number so that the monitoring may be performed in the requested quality from as low number of waypoints as possible), the latter maximizes the total coverage of the area of interest C (the goal is to find positions of N waypoints so that the visible area may be as large as possible). The third optimization criterion in Equation (12) is connected with the planning of routes R for individual UAVs (the goal is to find such routes so that the operation time T may be as short as possible):

(10) minimize(N)

(11) maximize(C)

(12) minimize(T)

The first two optimization criteria go against one another (the coverage is, generally, reduced when the number of waypoints is lowered). Thus, a new parameter called minimum coverage $C_{min}$ is defined; it controls the requested quality of the operation as it determines the minimum necessary portion of the visible area in the area of interest—see Equation (13). The objective is to find a low number of waypoints, with which the coverage is still equal or higher than requested:

(13) $$C\geq C_{min}$$

In general, each reconnaissance operation is characterized by a number of constant parameters and a number of optimization parameters (decision variables). Constant parameters are as follows:

- Geographical data: the terrain and database of obstacles in the area of operations (E, O).
- Size, shape and position of the area of interest (AoI).
- Number and basic positions of available UAVs, parameters of their sensors (M, U, $\alpha_{fov}$, $d_{max}$).
- Minimum and maximum permitted height of flight above the ground level ($h_{min}$, $h_{max}$).
- Minimum requested coverage ($C_{min}$).

Optimization parameters are as follows (all variables are continuous except the first one, which is discrete):

- Number of waypoints (N).
- Positions of waypoints (coordinates $x_{i}$ and $y_{i}$ for all $W_{i}\in W$).
- Heights above the ground level of the UAVs at waypoints ($h_{i}$ for all $W_{i}\in W$).

## 4. Solution Algorithms

In this section, algorithms for solving the problem formulated in the previous section are proposed. First, the principle for calculating the coverage of the area of interest is presented. Then, algorithms for the optimization of criteria in Equations (10)–(12) follow.

### 4.1. Evaluation of a Solution

Let $X^{N}$ be a particular solution, lying in the state space, to the reconnaissance operation. This solution is characterized by N waypoints W deployed in the area of operations: $X^{N}=\left\{W_{1},W_{2},\ldots ,W_{N}\right\}=\left\{x_{1},y_{1},h_{1},x_{2},y_{2},h_{2},\ldots ,x_{N},y_{N},h_{N}\right\}$, i.e., there are 3N optimization variables. For a particular problem (settings of constant parameters) and solution $X^{N}$ in the state space, the value of the coverage of the area of interest $C^{N}$ can be calculated: $C^{N}=f\left(X^{N}\right)$; the value lies in interval 〈0,1〉.

**Figure 5:**
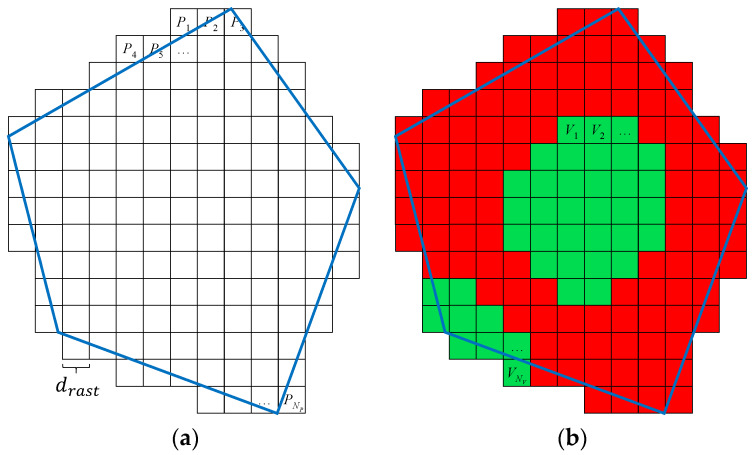
Rasterization of the area of interest: (**a**) points in the area of interest; (**b**) visible points.

For calculation of $C^{N}$, the number of visible points |V| in the area of interest needs to be determined—see Equation (3). To get this number, any point lying inside the area of interest must be evaluated if it is visible from any waypoint or not. In general, there are an infinite number of points *P* inside the area of interest, which is, of course, not possible to evaluate from the practical point of view. The rasterization of the area of interest is a possible solution to this. The principle is shown in Figure 5a. The area is evenly covered by a finite number of points P using the Sukharev grid. The rasterization step $d_{rast}$ determines the total number of points $N_{P}$; each point lies in the middle of its square. The visibility of each point in P is evaluated independently on one another; in this way, set V of visible points of size $N_{V}$ is created as illustrated in Figure 5b. The approximation of $C^{N}$ is then calculated according to Equation (14); the precision of the approximation can be controlled by the rasterization step $d_{rast}$:

(14) $$C^{N}=\frac{\left|V\right|}{\left|P\right|}=\frac{N_{V}}{N_{P}}$$

Evaluation of solution $X^{N}$ is performed by Algorithm 1. At the beginning, the set of visible points V is emptied (line 1), and set P is created based on the principles presented above (line 2). Then, each point $P_{j}\in P$ is evaluated independently (line 3 to 7); if point $P_{j}$ is evaluated as visible from at least one waypoint $W_{i}\in W$ (line 5), then this point is included in set V and the algorithm continues by evaluation of the next point. The algorithm returns the coverage $C^{N}$ of solution $X^{N}$ (lines 8 and 9).

**Algorithm 1** Evaluation of a solution in pseudocode

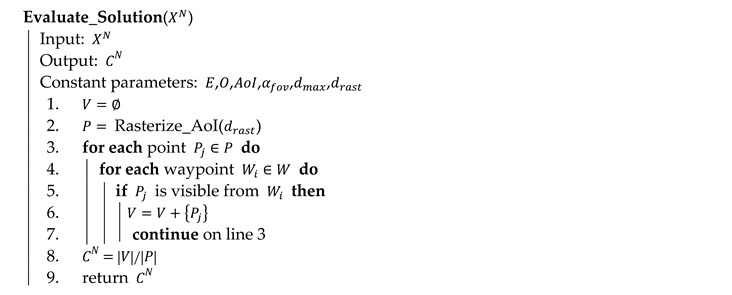



Algorithm 2 elaborates the key process on line 5 of Algorithm 1. It evaluates the visibility of point $P_{j}$ from waypoint $W_{i}$ by testing four conditions: (a) distance between $W_{i}$ and $P_{j}$ must not be larger than the maximum permitted distance $d_{max}$ (line 1); (b) $P_{j}$ must be in the field of view $\alpha_{fov}$ of the camera positioned in $W_{i}$ (line 2); (c) there is no obstacle $O_{k}\in O$ disrupting the VLOS between $W_{i}$ and $P_{j}$ (line 3 and 4); (d) there is no point $P_{k}\in P$ of elevation, which disrupts the VLOS between $W_{i}$ and $P_{j}$ (line 5 and 6).

**Algorithm 2** Visibility evaluation between a point and a waypoint in pseudocode

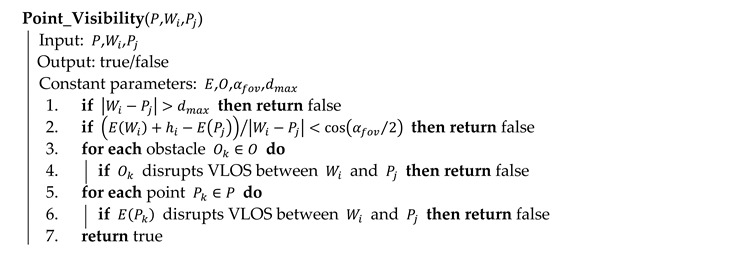



The computational complexity of Algorithm 1 is in Equation (15). The first and the second terms are related to lines 3 and 4 of Algorithm 1: the visibility needs to be evaluated between $N_{P}$ rasterized points and (up to) N waypoints. The third term represents the process on lines 3 and 4 of Algorithm 2: each obstacle from set O of size L may disrupt the VLOS. The last term is connected with lines 5 and 6 of Algorithm 2: elevation of points in P may disrupt the VLOS (the reason of square root of $N_{P}$ is that only points in P lying directly between $W_{i}$ and $P_{j}$ have to be tested):

(15) $$O\left(N_{P}\cdot N\cdot L\cdot \sqrt{N_{P}}\right)$$

The evaluation of a solution is a key process in the optimization of the problem. Therefore, the great efforts were devoted by the authors to increase its efficiency. Two main improvements were implemented: (a) the floating horizon algorithm for computing visibility of a set of points in a line with the linear dependence was implemented; (b) the obstacles were superimposed on the terrain. Thus, the resulting computational complexity of the improved algorithm was reduced as stated in Equation (16). Moreover, the implementation was optimized from the coding point of view (only operations with integers are employed in the critical parts of the algorithm). The process is also parallelized and distributed on the cores of a multicore processor:

(16) $$O\left(N_{P}\cdot N\right)$$

### 4.2. Optimization of Waypoint Deployment

In this section, an algorithm for optimization of a particular number of waypoints is proposed. The optimization criterion is to maximize the coverage $C^{N}$ according to Equation (11). The input of the algorithm, beside the constant parameters and algorithm settings, is the number on waypoints N to be deployed. The output is a particular solution $X^{N}=\left\{W_{1},W_{2},\ldots ,W_{N}\right\}=\left\{x_{1},y_{1},h_{1},x_{2},y_{2},h_{2},\ldots ,x_{N},y_{N},h_{N}\right\}$ and its coverage $C^{N}$.

The proposed metaheuristic algorithm is based on the simulated annealing principles, which are inspired in annealing in metallurgy (reducing defects of material by involving heating and controlled cooling). The algorithm works in iterations where the process of solution transformation is performed. The transformed solution may replace the original—even if it is worse. This principle allows expanding the search space and prevents the algorithm to be stuck in some local optimum.

The behavior or the algorithm is controlled by 5 parameters as follows:

- Maximum temperature $T_{max}$: the initial value of temperature used in the first iteration.
- Minimum temperature $T_{min}$: the threshold value of temperature (the algorithm ends when the temperature drops below this threshold).
- Cooling coefficient *λ*: it controls the speed of temperature reduction by cooling in successive iterations.
- Maximum number of transformations in iteration $n_{1max}$: it controls the higher limit of transformations performed per iteration.
- Maximum number of replacements in iteration $n_{2max}$: it controls the higher limit of replacements (accepting the transformed solution and replacing the original) per iteration.

Algorithm 3 shows the principles in pseudocode. At the beginning, a random solution is generated (line 1; see Algorithm 4 for details) and evaluated (line 2; see Algorithm 1 for details). The current temperature is set to the maximum limit $T_{max}$ (line 3). The algorithm works in iterations (lines 4 to 18); the temperature does not change within an iteration. In each iteration, a number of transformations are performed (lines 5 to 17). Transformation of the current solution $X^{N}$ into the new solution $X^{N^{\prime }}$(line 7) is the key process of the algorithm (see Algorithm 5 for details).

**Algorithm 3** Optimization of waypoint deployment in pseudocode

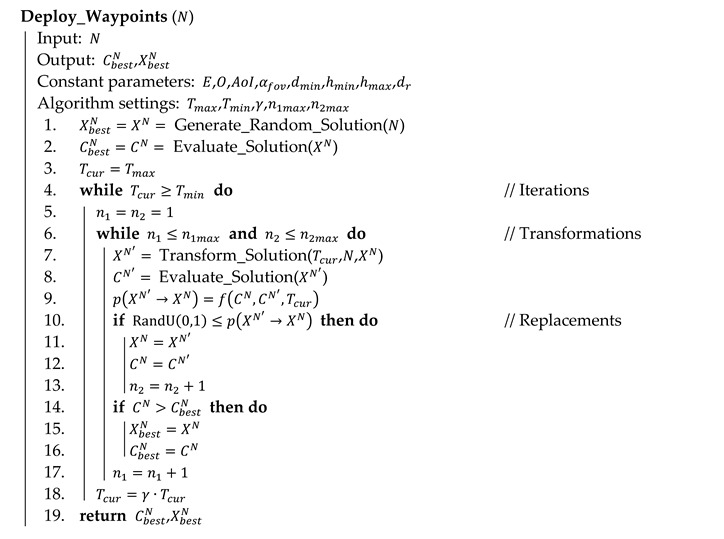



The original solution is replaced by the transformed solution with some probability (line 9) according to the Metropolis criterion in Equation (17). In case that the transformed solution is better than the original, the original is always replaced. Otherwise, the probability depends on the difference of qualities of both solutions and the current temperature $T_{cur}$: the lower the difference and the higher the temperature, the bigger the probability. This means that in the first phases of the algorithm, the state space is largely expanded by accepting often worse solutions, whereas, towards the end of the optimization, the solution is tuned in its surrounding. The acceptance of the transformed solution is decided on line 10; function RandU(0,1) is a pseudorandom number generator with a uniform distribution:

(17) $$p\left(X^{N^{\prime }}\to X^{N}\right)=\begin{array}{cc} \{\;\begin{array}{l} 1\\ e^{-\frac{C^{N}-C^{N^{\prime }}}{T_{cur}}} \end{array} & \begin{array}{l} \mathrm{for}\;C^{N^{\prime }}\geq C^{N}\\ \mathrm{otherwise} \end{array} \end{array}$$

An iteration ends when either the number of transformations $n_{1}$ or the number of replacements $n_{2}$ exceed their limits $n_{1max}$ and $n_{2max}$ respectively (see condition on line 6). Then the temperature is lowered (line 18) by cooling coefficient λ (0<λ<1) and the next iteration starts. The algorithm is terminated when the temperature $T_{cur}$ drops below its lower limit $T_{min}$ (see condition on line 4). The best solution found during the whole optimization $X_{best}^{N}$ is stored (lines 14 to 16) and returned at the end of the algorithm (line 19).

Algorithm 4 shows the process of the generation of a random solution (named on line 1 of Algorithm 3). Each variable of a solution is set in its permitted limits using a pseudorandom number generator with a uniform distribution (functions MinX, MaxX, MinY and MaxY return the minimum and maximum values of a circumscribed rectangle of the area of interest).

**Algorithm 4** Generation of a random solution in pseudocode

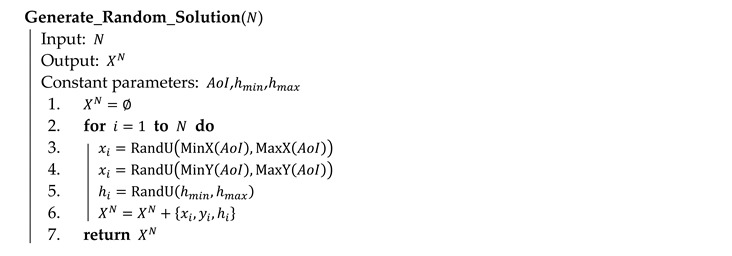



Algorithm 5 presents the process (called on line 7 of Algorithm 3), in which the current solution $X^{N}$ is transformed into the new solution $X^{N^{\prime }}$. In the transformation, one randomly selected variable is changed, the remaining variables copy the original values. The variable is selected by a pseudorandom integer number generator RandI(a,b) with a uniform distribution (line 2) in the range from 1 to 3N as the solution is composed of 3N variables: $X^{N}=\left\{x_{1},y_{1},h_{1},x_{2},y_{2},h_{2},\ldots ,x_{N},y_{N},h_{N}\right\}=\left\{X_{1}^{N},X_{2}^{N},\ldots ,X_{3N}^{N}\right\}$. The size of the change is determined by a pseudorandom number generator RandN(μ,σ) with a normal distribution with a mean of μ=0 and a standard deviation of σ calculated on line 6 according to Equation (18). The standard deviation depends on the current temperature and the range of the variable (calculated on lines 3, 4 and 5 for individual types of variables: x coordinate, y coordinate and height). The bigger the temperature, the bigger the changes. Constant ε in Equation (18) is a small number influencing the standard deviation in situations when the current temperature is close to its minimum limit:

(18) $$\sigma =\frac{\left(T_{cur}-T_{min}\right)\cdot \left(\frac{range}{3}-\varepsilon \right)}{T_{max}-T_{min}}+\varepsilon$$

The computational complexity of the algorithm for optimizing waypoint deployment is given in Equation (19). It is linearly dependent on the number of iterations $n_{iter}$ computed according to Equation (20) and the maximum number of transformations $n_{1max}$ ($n_{1max}$ is used instead of $n_{2max}$ because the number of transformations is always equal to or greater than the number of replacements). The last two terms correspond to the evaluation of the transformed solution in the loop—see Equation (16):

(19) $$O\left(n_{iter}\cdot n_{1max}\cdot N_{P}\cdot N\right)$$

(20) $$n_{iter}=\lceil \frac{\log T_{min}-\log T_{max}}{\log\gamma }\rceil$$

The algorithm was implemented in two versions: continuous and discrete. The former assumes all the variables of the solution to be continuous—waypoints can be deployed wherever in the area of operations. The latter one assumes the variables to be discreet with the distance between neighboring values set to the size of the rasterization step $d_{rast}$. It has an effect that the waypoints can be deployed only in the middle of rasterization squares. The discretization of each variable is performed by rounding to the nearest permitted value.

**Algorithm 5** Transformation of a solution in pseudocode

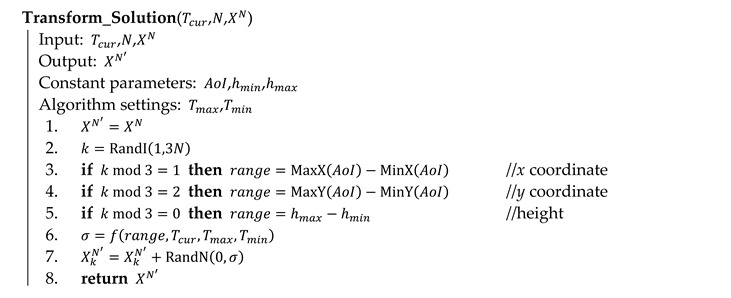



### 4.3. Optimizing the Number of Waypoints

This section presents Algorithm 6 for optimizing the number of waypoints according to the optimization criterion in Equation (10). The main idea of the algorithm consists in the first estimation of the number based on the parameters of sensors and the subsequent deployment of this number of waypoints; in the next phases, the value is updated according to the gap between the actual and required coverage. Of course, this is not the only approach to determine the number of waypoints. Another approach could be the binary search method where the value is determined between the limits by the interval halving. The algorithm proposed in this section was selected because of its low number of phases (see the results in Section 5.3).

**Algorithm 6** Optimizing the number of waypoints in pseudocode

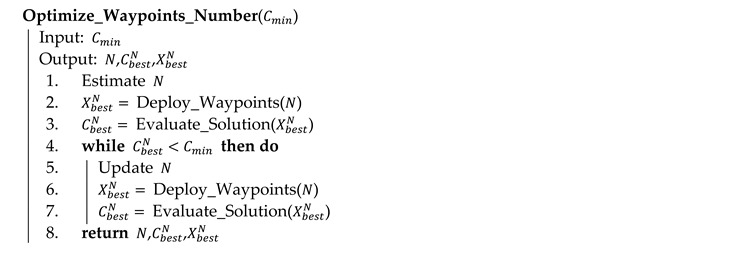



The input of the algorithm is the minimum required coverage $C_{min}$. At first, the initial value of N using Equation (21) is estimated (line 1) based on the size of the area of interest and parameters of sensors. Coefficient τ≥1 is a constant, which can be set with regard to the parameters of scenarios (e.g., the terrain and obstacles); in most real situations, the best results were achieved with τ=1.5 (the purpose of this coefficient is discussed in Section 5.3 in more detail). Then, N waypoints are optimized using Algorithm 3 (lines 2 and 3):

(21) $$N=\lceil \frac{\tau \cdot C_{min}\cdot \left|AoI\right|}{\pi \cdot {\left(d_{max}\cdot \sin\frac{\alpha_{fov}}{2}\right)}^{2}}\rceil$$

If the coverage does not satisfy the minimum limit (line 4), the value of N is updated (line 5) according to Equation (22) and the new number of waypoints are deployed again using Algorithm 3 (lines 6 and 7). This process is repeated in a loop (lines 4 to 7) until such N is found that the minimum coverage constraint is met. The algorithm returns the number N as well as the solution $X^{N}$ and its quality $C^{N}$ (line 8):

(22) $$N=\lceil N\cdot \frac{C_{min}}{C^{N}}\rceil$$

### 4.4. Planning of Routes

When the number of waypoints and their deployment in the area of operations are determined, the problem of planning routes for available UAVs follows. The optimization criterion is to minimize the duration T of the reconnaissance operation as stated in Equation (12). This problem can be easily transformed into the well-known Min-Max Multi-Depot Vehicle Routing Problem (MDVRP) [35] where a set of customers should be served by a fleet of vehicles originating from multiple depots.

The authors of this article studied this problem extensively in their previous research. The metaheuristic algorithm based on the Ant Colony Optimization (ACO) theory in combination with other principles was proposed as a solution. The results were published, for example, in [36,37]. Therefore, this topic will not be examined and further pursued in this article.

## 5. Experiments and Results

The solutions proposed in the previous section were verified on a series of experiments. All experiments were carried out on a computer with configuration as follows: CPU Intel i7-7700 3.5 GHz (4 cores), 32 GB RAM.

### 5.1. Evaluation of a Solution

The first set of experiments is aimed at the algorithm for the evaluation of a solution, i.e., calculation of the coverage for a particular solution, with regard to its efficiency and influence of the key parameters: number of waypoints and the total number of points in the area of interest.

**Figure 6:**
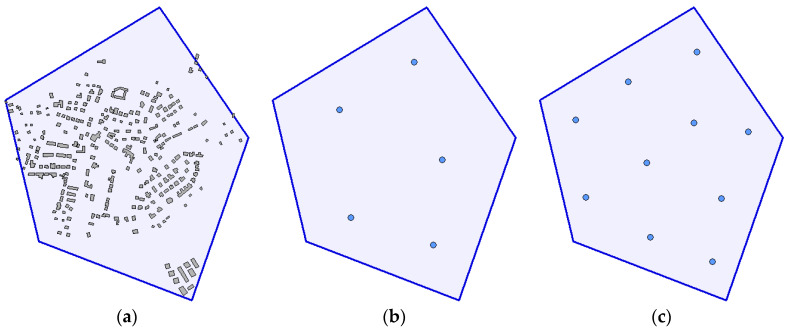
Scenario for the evaluation of a solution: (**a**) area of interest and the layout of obstacles; (**b**) deployment of waypoints for N=5; (**c**) deployment of waypoints for N=10.

The constant parameters of the scenario for these experiments are as follows: number of waypoints N=5 and N=10, respectively, field of view of sensors $\alpha_{fov}=90{}^{\circ}$, maximum permitted distance between a camera and objects $d_{max}=240\;m$, minimum and maximum height of flight $h_{min}=50\;m$ and $h_{max}=300\;m$, width and height of the area of interest 950 m × 1150 m, number of obstacles in the area of interest L=266 (height of obstacles ranges between 5 m and 15 m and their average height is 6.4 m), the terrain relatively flat (the difference between the minimum and maximum altitude in the area of operations is about 80 m). The shape of the area of interest and the layout of obstacles are shown in Figure 6a. Figure 6b,c show positions of deployed waypoints.

Table 1 and Table 2 show the results of the experiments. Table 1 presents the results achieved using the original algorithm with no code optimization (see Section 4.1) and the computational complexity given by Equation (15). In Table 2, the results are achieved when using the improved (optimized) algorithm with the computational complexity from Equation (16).

In general, the original algorithm can be assumed more precise in evaluation than the optimized version. In order to increase its efficiency, the latter one works with the integers rather than real numbers in its critical sections; this may lead to minor approximations and inaccuracies. To calculate the coverage error, the solution achieved by the original algorithm for $d_{r}=1$ is used as benchmark (both for N=5 and N=10).

The difference in the execution time (time to evaluate a solution) is immense. The main reason is repeating the VLOS tests of each point with a number of *L* obstacles in the original algorithm (see Equation (15)), while this is not done in the optimized version (see Equation (16)). In average (based on all experiments in Table 1 and Table 2), the optimized version is almost 60 times faster than the original algorithm. For example, in case of  N=5 and $d_{r}=1$, the optimized algorithm manages to evaluate more than 30 million points per second, whereas the original version evaluates only 0.5 million points.

**Figure 7:**
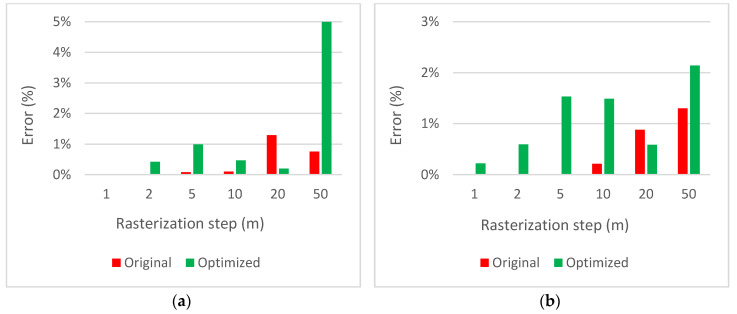
Coverage errors for the original and optimized versions of the algorithm for the solution evaluation: (**a**) number of waypoints N=5; (**b**) number of waypoints N=10.

**Figure 8:**
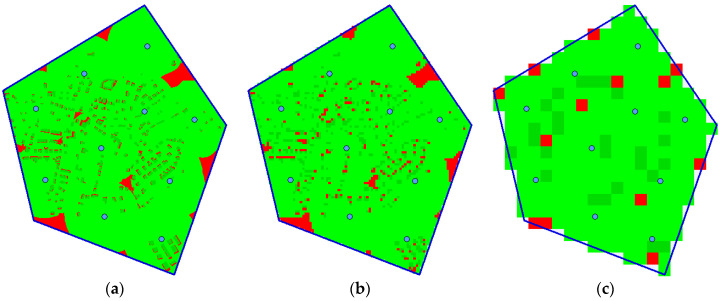
Influence of the size of the rasterization step on the solution evaluation: (**a**) rasterization step $d_{rast}=1$; (**b**) rasterization step $d_{rast}=10$; (**c**) rasterization step $d_{rast}=50$.

Figure 7 compares the coverage errors. Although the original version provides more precise results than the optimized version, the error of the latter one is below 2% in all cases (with the exception of $d_{rast}=50$, but the reduction of information is too big in this case). The illustration of the influence of the rasterization step on the evaluation is shown in Figure 8 for rasterization steps $d_{rast}=1$, $d_{rast}=10$ and $d_{rast}=50$.

**Figure 9:**
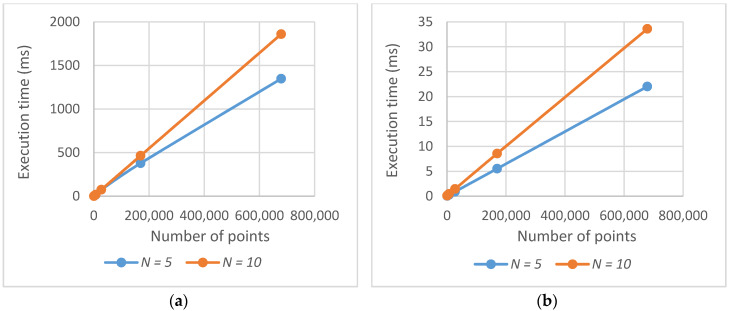
Dependence of the execution time on the number of points: (**a**) original algorithm; (**b**) optimized algorithm.

Figure 9 shows the dependence of the execution time on the number of points $N_{P}$ to be evaluated in a solution (for the original algorithm on the left, for the optimized version on the right). The linear dependence is apparent. The linear dependence on the number of waypoints *N* can also be seen in the graphs. However, it is not that twice the number of waypoints means twice the execution time (it is only about 1.6 times in this case). The reason is that when a point is once evaluated as visible, it does not have to be evaluated again from other waypoints; this case is more frequent when there are more waypoints.

### 5.2. Optimizing the Waypoint Deployment

A set of experiments was designed to validate the algorithm proposed in Section 4.2 for the deployment of a number of waypoints in the area of operations. Conditions and parameters for the benchmark scenarios are as follows:

- The area of interest is assembled by joining a number of hexagons with the circumradius 100 m.
- The terrain is absolutely flat (the altitude does not change within the area of operations).
- There are no obstacles in the area of operations.
- Parameters of sensors are $d_{max}=\sqrt{2}\cdot 100$, $\alpha_{fov}=90{}^{\circ}$.
- The number of waypoints to be deployed are the same as the number of hexagons.

**Figure 10:**
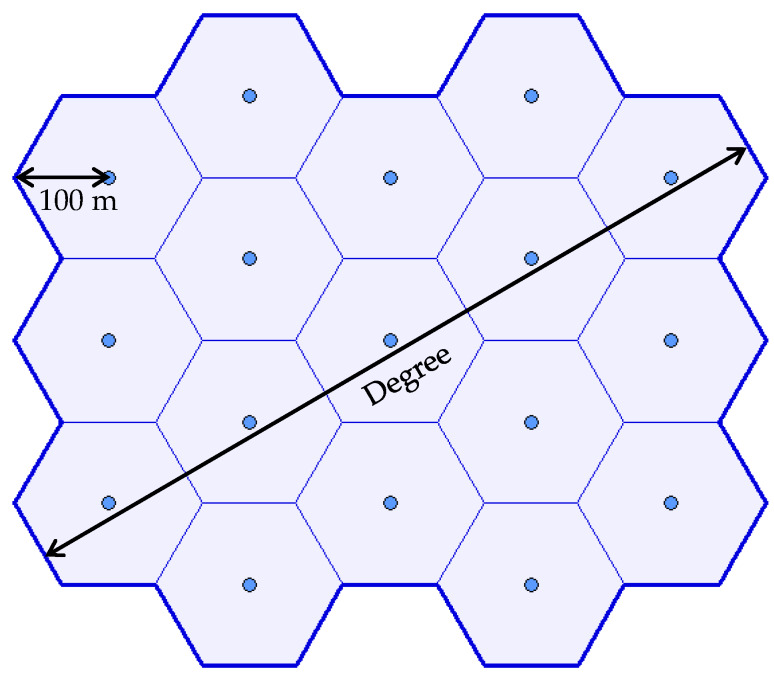
Area of interest created by joining hexagons.

The principle of creating the area of interest is presented in Figure 10. The degree determines the number of hexagons in the diagonal. In the example, the degree equals 5, which means that 17 hexagons were joined to create the area of interest and the same number of waypoints N=17 are to be deployed.

This principle along with the conditions mentioned above ensures that the optimal solution can be easily found. When the waypoints are deployed optimally, the whole area of interest will be covered ($C_{\%}^{N}=100\%$). The optimal solution is as follows:

- The waypoints (coordinates $x_{i}$, $y_{i}$) lie in the centers of the hexagons (see Figure 10).
- The monitoring height is exactly 100 m above the ground level ($h_{i}=100\;m$).

Six benchmark instances were created for verification. They differ by the degree as shown in Table 3. The fifth column of Table 3 shows the number of waypoints and the last column the number of variables per a solution vector $X^{N}$. For example, in the case of instance d06, the solution is composed of more than 200 independent variables. The permitted range of the flight height does not differ in the instances.

Both the continuous and discrete versions of the algorithm proposed in Section 4.2 (Algorithm 3) were used to find the solutions of the benchmark problems and the results were compared with the optimal solutions. The parameters of the algorithm were set as follows: $T_{max}=10^{-2}$, $T_{min}=10^{-6}$, γ=0.9, $n_{1max}=200$, $n_{2max}=20$. The algorithm was executed 50 times on each benchmark instance.

The results achieved with the continuous version of the algorithm are recorded in Table 4. The optimal solution was found in the case of the simplest instances (d01 and d02). In other instances, a solution was found very close to the optimum. The difference between the best solution and the optimum (referred to as error in Table 4) is below 2% in all cases. The execution time depends on the rasterization step $d_{rast}$.

Table 5 shows the results achieved by the discrete version of the algorithm. The optimum was found in case of instances d01, d02, d03; the maximum error in case of the most complex problems is below 1%. The execution time is slightly higher than in case of the continuous version; this is caused by the discretization of the variables in the transformation process.

**Figure 11:**
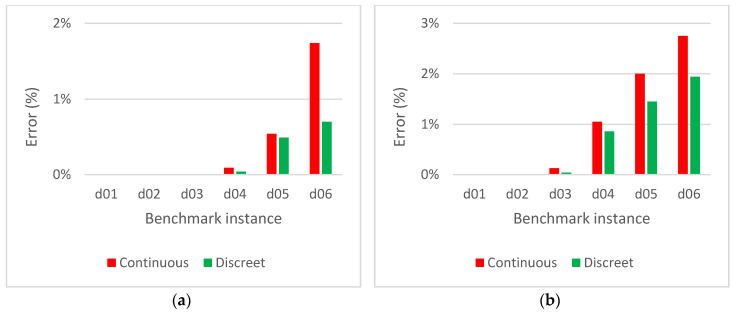
Comparison of the continuous and discrete versions of the algorithm: (**a**) error of the best solutions; (**b**) error of the average values.

**Figure 12:**
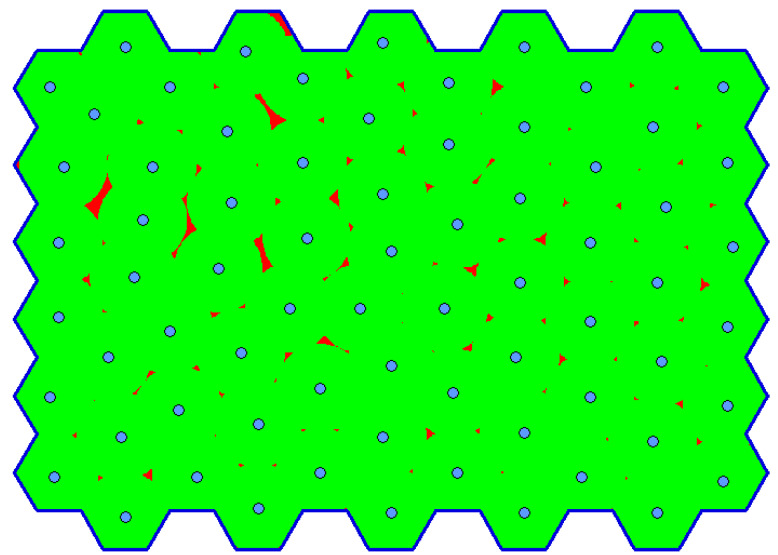
The best solution found for instance d06.

The comparison of the results achieved by the continuous and discrete versions of the algorithm is shown in Figure 11. In general, the discrete version provides better solutions than the continuous version; the more complex the problem, the better the results obtained by the discrete version. Therefore, the discrete version is used further on. Figure 12 shows the best solution achieved by the discrete version of the algorithm for instance d06 ($C_{\%}^{71}=99.30\%$).

### 5.3. Optimizing the Number of Waypoints

In this section, the algorithm proposed for optimizing the number of waypoints (Algorithm 6) is verified on the benchmark problems. The same problems as proposed in the previous section are used.

The algorithm starts with the first estimation of N according to Equation (21). The part of this equation is coefficient τ. This coefficient takes specific parameters and characteristics of the environment into consideration (e.g., the terrain and the number of obstacles). In the ideal scenario, where the waypoints could be deployed in such a way that the whole area would be covered from a number of waypoints and, at the same time, areas visible from sensors would not overlap, the coefficient should be τ=1 and the estimation would correspond to the correct number. In the benchmark problems proposed for verification, where the terrain is flat with no obstacles, the waypoints could be deployed so that the visible areas might overlap just slightly; therefore, the coefficient was set to τ=1.1. In the real scenarios, the best value of the coefficient was empirically found as τ=1.5.

**Figure 13:**
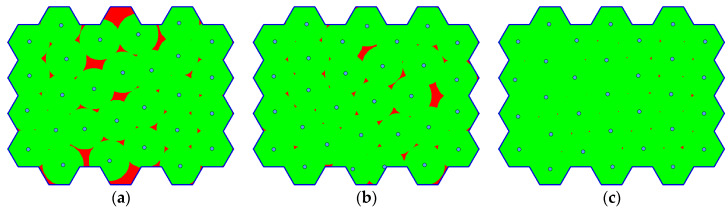
The progress in optimizing the number of waypoints, for instance d04: (**a**) first estimation (N=28); (**b**) first update (N=30); (**c**) second update (N=31).

Table 6 shows the results of the algorithm. The minimal coverage is set to $C_{min}=99\%$. The reason for this is that the full coverage represents the optimal solution, which can be hardly achieved in case of the more complex problems. Therefore, the algorithm error of 1% is assumed. As can be seen, the optimal numbers of waypoints are estimated in case of all the benchmark instances. Moreover, it was done in at most 3 phases: the first estimation (line 1 of Algorithm 6) and 2 updates (line 5 of Algorithm 6). Figure 13 illustrates the situation in case of instance d04.

### 5.4. Experiments on Real Scenarios

The experiments in this section are based on the real scenarios that reflect typical reconnaissance operations. The environment of the scenarios is based on the real geographic data using two models: (a) The Digital Elevation Model (DEM), and (b) The Topographic Digital Data Model (TDDM). The former is a representation of the terrain surface in the form of a heightmap. The latter is a database of topographic and other objects; in the scenarios, buildings are used as not-transparent obstacles.

Both geographic models come from the Military Geographic and Hydrometeorologic Office of the Ministry of Defense of the Czech Republic which provides geographic data for the Army of the Czech Republic. The DEM model has, in its last version, the distance between elevations 2.5 m and elevation precision 0.3 m; it is being updated regularly by methods of digital stereophotogrammetry and airborne laser scanning. The TDDM model contains topographic objects represented by a polygon and parameters (e.g., object height); it is being regularly updated using the method of direct mapping with the support of aerial imaging.

Table 7 characterizes the environment of the scenarios: the size of the area of interest, the elevation difference (difference between the highest and the lowest altitude inside the area of interest) and obstacles (their number and average height). The scenarios offer various types of environment: from a small to large area to be explored, from a relatively flat to a very uneven terrain, from a low density to a high density of obstacles. For example, scenario sc04 is a typical urban environment with an irregular shape of the area of interest, a very high density of tall obstacles (buildings) with narrow streets and a flat terrain. In comparison with this configuration, scenario sc05 is a large mountain environment with a very low density of obstacles, but a very uneven terrain.

Table 8 presents the technical and tactical configurations: the number of UAVs available (at the disposal of the commander and deployed in the area of operations), the minimum requested coverage, parameters of sensors (the maximum distance to objects of interest and an angular field of view) and a minimum and maximum permitted height of flight of the UAVs.

For optimization, the parameters of the algorithms were set as follows:$\;T_{max}=10^{-2}$, $T_{min}=10^{-6}$, γ=0.9, $n_{1max}=10\cdot n_{2max}$, $n_{2max}=20$ (for sc01, sc02, sc05) or $n_{2max}=50$ (sc03, sc04) respectively.

Table 9 presents the optimization results. The third column shows the number of waypoints estimated by Algorithm 6. The estimated number of waypoints were deployed using Algorithm 3; in total, 50 optimizations per scenario were conducted—for results, see the fourth to sixth columns. The best solution found exceeds the minimum coverage required for the scenarios (see Table 8) in all cases. The last column shows the average execution time of optimization; this depends, of course, on the size of the rasterization step.

**Figure 14:**
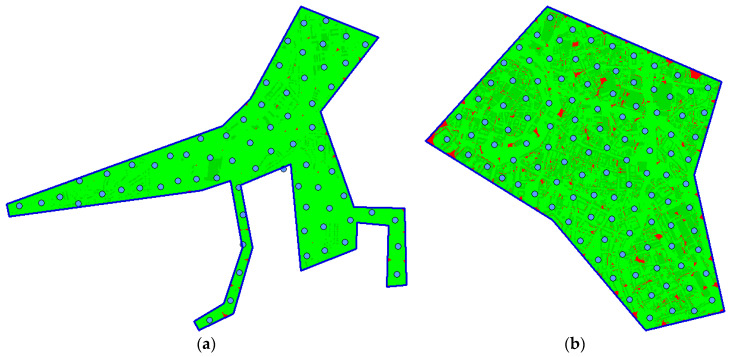
The best solution found: (**a**) instance sc03; (**b**) instance sc04.

Figure 14 illustrates the deployment of waypoints and coverage for scenarios sc03 (on the left) and sc04 (on the right). In case of the former one, the area of interest is of the very irregular shape. Despite of this, the algorithm managed to deploy all the waypoints inside the area (70 waypoints means 210 variables in the solution vector). In case of the latter one, the solution vector is composed of more than 300 independent variables.

For the best solutions found, the routes of UAVs available in individual scenarios were planned using the algorithm mentioned in Section 4.4. The results are recorded in Table 10. The last two columns show the parameters of the optimized routes: the total distance covered by all UAVs participating in the operation, and operation time T (the time when all the UAVs are back in their base positions); the latter is the optimization criterion defined in formula (12). In all cases, UAVs with the homogeneous flight parameters were used; the average flight velocity was set to $10\;m\cdot s^{-1}$.

**Figure 15:**
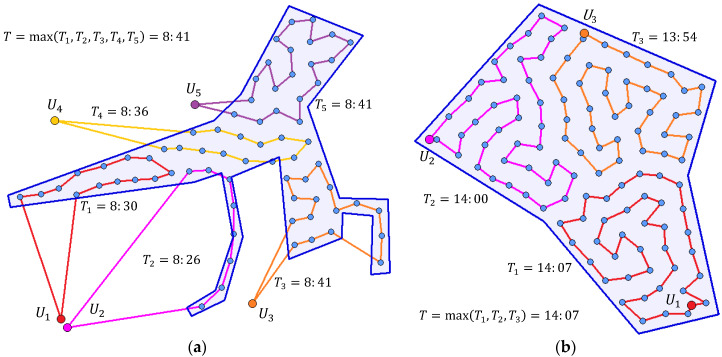
Planning the routes: (**a**) instance sc03; (**b**) instance sc04.

Figure 15 shows the routes for scenarios sc03 (on the left) and sc04 (on the right). The routes of individual UAVs are color coded. Moreover, the route times (times needed for UAVs to conduct their routes) are stated. The operation ends when the last UAV returns back to its base; thus, the optimization is about a good distribution of waypoints to available UAVs. The similar times of individual routes are apparent.

The routes for UAVs are created by connecting the waypoints by straight lines (see Figure 15). This means that the routes are easily applicable for rotary-wing aircraft with the ability of vertical take-off and landing (VTOL) and abrupt changes in direction. The only requirement for them is the ability to automatically follow a set of predefined waypoints (and, of course, it needs to be equipped with an appropriate sensor). Nowadays, this ability is common not only for the state-of-the-art military UAVs but also for ordinary commercial drones. However, the model is applicable, with some minor limitations, even for fixed-wing aircraft—see [38] for more details.

The experiments conducted so far have assumed that the monitoring can be performed only when the UAVs are located at waypoints, i.e., not during their flight between them. In many real situations, the monitoring can be performed continuously during the flight of the UAVs along their routes. This case was tested on the best solutions found (Table 9) and the routes planned for these solutions (Table 10). The results are in Table 11 where the original coverage (monitoring only from waypoints) is compared to the so-called continuous coverage (monitoring during the flight). The continuous monitoring provides better results (the worse original solution, the higher the improvement).

## 6. Conclusions

The article presented the model of aerial reconnaissance using UAVs cooperating to conduct the operation as effectively as possible. The monitoring is performed from a number of waypoints deployed in the three-dimensional space in the area of operations. The terrain and non-transparent objects, which may cause occlusion, are taken into account. For the deployment of waypoints, the metaheuristic algorithm was proposed and verified by experiments; the results were compared with the optimal solutions. The possibility of the practical use was confirmed by a set of experiments based on the typical military reconnaissance scenarios and real geographic data.

The metaheuristic algorithm proposed for waypoints deployment is based on the simulated annealing principles. It is a simple algorithm yet very efficient in positioning problems. The strengths of the algorithm are its fast convergence to the optimum, efficient mechanism preventing a solution being stuck in some local optimum and low memory demands. The most time-consuming and memory demanding part of the whole optimization process is the evaluation of a particular solution. Therefore, a lot of effort was put into increasing its efficiency. The speed of the solution evaluation, as well as the memory demands, depends, beside the configuration of a computer used (power and available memory), on the rasterization; the number of points needed to be evaluated changes quadratically with the rasterization step. However, the precision of the evaluation is also dependent on the rasterization step. This results in two contrary requirements. On the one hand, the step should be as small as possible so that the evaluation is as precise as possible; on the other hand, it should be as large as possible so that the evaluation is as fast as possible. The choice of the rasterization step is, therefore, a compromise between the two requirements.

The model proposed in this article was implemented into the decision support system for commanders of the Czech Army. Prior to any military mission there must be planning process which is called either Troops leading procedure (in case of company and below military units) or Military decision making process (in case of battalion level and higher). This process is complex, continuous and composed of many steps. As it is apparent from the results of experiments, time of optimization did not exceed 15 min in case of the most complex problems. During this time, there are other activities which are ongoing simultaneously. Moreover, ordinary planning process with no autonomous computation must be done by military personnel and lasts for significantly longer time. In conclusion, time of computation did not influence any critical military mission at all.

Although the model is intended to be used for military purposes, there are other civil real life implementations and applications such as the rapid evaluation of a disaster area and management of recovery resources, identification of threats on land, border surveillance and protection, etc.

The future work of the authors will be aimed at further optimization of the solution evaluation process. The significant speed improvement is to be achieved by implementing the algorithm on the graphics processing unit (GPU). For the further verification of the model, the experiments using the real UAVs are planned to be carried out. The current reconnaissance model will also be extended to the persistent surveillance model, in which the continuous monitoring of the area of interest by a swarm of UAVs is assumed.

## Figures and Tables

**Table 1:** Experiments with the solution evaluation via the original algorithm.

Number of Waypoints N	$\mathrm{Rasterization}\;\mathrm{Step}\;d_{rast}(m)$	$\mathrm{Number}\;\mathrm{of}\;\mathrm{Points}\;N_{P}$	$\mathrm{Coverage}\;C^{N}$	Coverage Error	Execution Time (ms) ^1^
5	1	678,953	59.49%	Benchmark	1347
5	2	169,740	59.49%	0.00%	378
5	5	27,160	59.54%	0.08%	74
5	10	6786	59.43%	0.10%	15
5	20	1696	60.26%	1.29%	4.4
5	50	271	59.04%	0.76%	0.7
10	1	678,953	93.98%	Benchmark	1859
10	2	169,740	93.98%	0.00%	465
10	5	27,160	93.97%	0.01%	75
10	10	6786	93.78%	0.21%	18
10	20	1696	94.81%	0.88%	5.1
10	50	271	95.20%	1.30%	1.3

^1^ Each experiment was performed 1000 times and results were averaged.

**Table 2:** Experiments with the solution evaluation via the optimized algorithm.

Number of Waypoints N	$\mathrm{Rasterization}\;\mathrm{Step}\;d_{rast}\;(m)$	$\mathrm{Number}\;\mathrm{of}\;\mathrm{Points}\;N_{P}$	$\mathrm{Coverage}\;C^{N}$	Coverage Error	Execution Time (ms) ^1^
5	1	678,953	59.50%	0.02%	22.02
5	2	169,740	59.74%	0.42%	5.53
5	5	27,160	60.08%	0.99%	0.92
5	10	6786	59.77%	0.47%	0.28
5	20	1696	59.37%	0.20%	0.11
5	50	271	56.52%	4.99%	0.06
10	1	678,953	94.19%	0.22%	33.58
10	2	169,740	94.54%	0.60%	8.56
10	5	27,160	95.42%	1.53%	1.45
10	10	6786	95.38%	1.49%	0.47
10	20	1696	94.53%	0.59%	0.19
10	50	271	91.97%	2.14%	0.11

^1^ Each experiment was performed 1000 times and results were averaged.

**Table 3:** Benchmark instances for verification of the deployment algorithm.

Benchmark Instance	$\mathrm{Minimum}\;\mathrm{Height}\;h_{min}$	$\mathrm{Maximum}\;\mathrm{Height}\;h_{max}$	Degree	Number of Waypoints N	$\mathrm{Number}\;\mathrm{of}\;\mathrm{Variables}\;\mathrm{in}\;X^{N}$
d01	50 m	150 m	1	1	3
d02	50 m	150 m	3	7	21
d03	50 m	150 m	5	17	51
d04	50 m	150 m	7	31	93
d05	50 m	150 m	9	49	147
d06	50 m	150 m	11	71	213

**Table 4:** Results for benchmark problems achieved by the continuous version of the algorithm.

Instance	$\mathrm{Rast}.\;\mathrm{Step}\;d_{rast}(m)$	Optimal Solution	Solution Found ^1^			Error ^2^	Execution Time (s) ^1^
			Best	Mean	Stdev		
d01	2	100%	100%	100%	0.00%	0.00%	2.9
d02	2	100%	100%	100%	0.00%	0.00%	31.4
d03	5	100%	99.99%	99.87%	0.51%	0.01%	16.8
d04	5	100%	99.91%	98.95%	0.84%	0.09%	32.4
d05	10	100%	99.46%	98.00%	0.58%	0.54%	19.6
d06	10	100%	98.26%	97.25%	0.46%	1.74%	28.1

^1^ 50 trials. ^2^ Difference between the best and optimal solution.

**Table 5:** Results for benchmark problems achieved by the discrete version of the algorithm.

Instance	$\mathrm{Rast}.\;\mathrm{Step}\;d_{rast}(m)$	Optimal Solution	Solution Found ^1^			Error ^2^	Execution Time (s) ^1^
			Best	Mean	Stdev		
d01	2	100%	100%	100%	0.00%	0.00%	3.2
d02	2	100%	100%	100%	0.00%	0.00%	32.1
d03	5	100%	100%	99.96%	0.02%	0.00%	18.2
d04	5	100%	99.96%	99.14%	0.67%	0.04%	34.5
d05	10	100%	99.51%	98.55%	0.43%	0.49%	21.0
d06	10	100%	99.30%	98.06%	0.45%	0.70%	31.0

^1^ 50 trials. ^2^ Difference between the best and optimal solution.

**Table 6:** Results of the algorithm for the optimizing the number of waypoints.

Instance	Optimal N	First Estimation		First Update		Second Update	
		$N_{1}$	$C_{\%}^{N_{1}}$	$N_{2}$	$C_{\%}^{N_{2}}$	$N_{3}$	$C_{\%}^{N_{3}}$
d01	1	1	100%	–	–	–	–
d02	7	7	100%	–	–	–	–
d03	17	16	96.90%	17	99.96%	–	–
d04	31	28	94.77%	30	97.72%	31	99.88%
d05	49	45	95.67%	48	98.35%	49	99.13%
d06	71	65	95.68%	68	96.98%	71	99.03%

**Table 7:** Characteristics of the scenario’s environment.

Scenario	Area of Interest			Elevation Difference	Obstacles	
	Width	Height	Area		Count	Height
sc01	0.40 km	0.25 km	0.1 km^2^	44 m	14	14.6 m
sc02	0.95 km	1.14 km	0.7 km^2^	79 m	266	6.4 m
sc03	3.75 km	3.04 km	2.8 km^2^	129 m	533	5.1 m
sc04	2.99 km	3.24 km	5.3 km^2^	47 m	936	10.6 m
sc05	4.00 km	2.10 km	6.8 km^2^	654 m	8	9.4

**Table 8:** Technical and tactical parameters of the reconnaissance operations.

**Scenario**	**Number of UAVs**	**Minimum Coverage**	**Sensors**		**Minimum Height**	**Maximum Height**
			$d_{max}$	$\alpha_{fov}$		
sc01	1	99%	80 m	75°	20 m	120 m
sc02	2	98%	150 m	75°	20 m	180 m
sc03	5	99%	200 m	90°	50 m	300 m
sc04	3	95%	200 m	90°	50 m	300 m
sc05	4	99%	500 m	120°	50 m	400 m

**Table 9:** Optimizations of the number and deployment of waypoints for the real scenarios.

Scenario	$\mathrm{Rast}.\;\mathrm{Step}\;d_{rast}(m)$	Number of Waypoints	Solution Found ^1^			Execution Time (s) ^1^
			Best	Mean	Stdev	
sc01	2	23	99.19%	98.09%	1.06%	22.9
sc02	3	37	98.58%	98.05%	0.28%	83.1
sc03	5	70	99.26%	98.68%	0.41%	770.4
sc04	5	111	95.47%	94.88%	0.26%	772.8
sc05	10	22	99.03%	98.21%	0.38%	66.7

^1^ 50 trials.

**Table 10:** Optimizing the routes.

Scenario	Number of UAVs	Number of Waypoints	Coverage	Routes	
				Distance (km)	Time T (min)
sc01	1	23	99.19%	1.515	2:31
sc02	2	37	98.58%	6.823	5:41
sc03	5	70	99.26%	25.756	8:41
sc04	3	111	95.47%	25.234	14:07
sc05	4	22	99.03%	16.067	7:11

**Table 11:** Comparison of the original and continuous monitoring.

Scenario	Number of UAVs	Number of Waypoints	Waypoints Coverage	Continuous Coverage	Improvement
sc01	1	23	99.19%	99.71%	0.52%
sc02	2	37	98.58%	99.50%	0.92%
sc03	5	70	99.26%	99.66%	0.40%
sc04	3	111	95.47%	97.82%	2.35%
sc05	4	22	99.03%	99.70%	0.67%

## Nomenclature

AoO	Area of operations.
AoI	Area of interest to be explored.
U	Set of available unmanned aerial vehicles: $U=\left\{U_{1},U_{2},\ldots ,U_{M}\right\}$.
$U_{i}$	An available UAV.
M	Number of available unmanned aerial vehicles.
W	Set of waypoints deployed in the area of operations: $W=\left\{W_{1},W_{2},\ldots ,W_{N}\right\}$.
$W_{i}$	A waypoint deployed in the area of operations.
$x_{i}$,$y_{i}$	Coordinates of waypoint $W_{i}$ in the area of operations.
N	Number of waypoints deployed in the area of operations.
$\alpha_{fov}$	Angular field of view of sensors of the UAVs.
$d_{max}$	Maximum distance from a sensor to an object of interest.
$h_{alt}^{i}$	Altitude of an UAV at waypoint $W_{i}\in W$.
$h_{i}$	Height of flight of an UAV above the ground level at waypoint $W_{i}\in W$.
$h_{min}$	Minimum allowed height of flight of UAVs.
$h_{max}$	Maximum allowed height of flight of UAVs.
P	Set of points on the ground in the area of operations: $P=\left\{P_{1},P_{2},\ldots \right\}$.
E	Function to determine the terrain elevation in any point in the AoO.
O	Set of obstacles in the area of interest: $O=\left\{O_{1},O_{2},\ldots ,O_{L}\right\}$.
$O_{k}$	An obstacle in the area of interest.
L	Number of obstacles in the area of interest.
$V_{i}$	Set of points in the area of interest visible from waypoint $W_{i}\in W$.
V	Set of points in the area of interest monitored from all the waypoints.
$d_{rast}$	Size of the rasterization step.
$N_{P}$	Total number of points in the rasterized area of interest.
$N_{V}$	Number of visible points in the rasterized area of interest.
$X^{N}$	Solution in the state space (particular deployment of waypoints).
C,$C^{N}$	Coverage of the area of interest from all the waypoints.
$C_{min}$	Minimum required coverage of the area of interest.
R	Routes of UAVs: $R=\left\{R_{1},R_{2},\ldots ,R_{M}\right\}$.
$R_{j}$	A route of UAV $U_{j}$. (order of nodes to be visited): $R_{j}=\left\{R_{j}^{0},R_{j}^{1},R_{j}^{2},\ldots ,R_{j}^{K_{j}},R_{j}^{K_{j}+1}\right\}$.
$K_{j}$	Number of waypoints to be visited en route $R_{j}$.
$T_{j}$	Time to perform route $R_{j}$ by $U_{j}$.
T	Duration of the reconnaissance operation.
$T_{max}$	Initial temperature (simulated annealing parameter).
$T_{min}$	Minimum temperature threshold (simulated annealing parameter).
$T_{cur}$	Current temperature (variable used in the simulated annealing algorithm).
γ	Cooling coefficient (simulated annealing parameter).
$n_{1max}$	Number of transformations (simulated annealing parameter).
$n_{2max}$	Number of replacements (simulated annealing parameter).
τ	Constant used when estimating the necessary number of waypoints.
μ	Mean.
σ	Standard deviation.
ε	Constant used in the solution transformation process.
